# Computer aided diagnosis of neurodevelopmental disorders and genetic syndromes based on facial images – A systematic literature review

**DOI:** 10.1016/j.heliyon.2023.e20517

**Published:** 2023-10-05

**Authors:** Fábio Rosindo Daher de Barros, Caio Novais F. da Silva, Gabriel de Castro Michelassi, Helena Brentani, Fátima L.S. Nunes, Ariane Machado-Lima

**Affiliations:** a School of Arts, Sciences and Humanities – University of Sao Paulo (USP), Av. Arlindo Bettio, 1000, Sao Paulo, 03828-000, Sao Paulo, Brazil; b Department of Psychiatry, University of Sao Paulo's School of Medicine (FMUSP), Sao Paulo, 05403-903, Sao Paulo, Brazil

**Keywords:** Machine learning, Computer-aided diagnosis, Facial dysmorphism, Facial classification, Deep learning

## Abstract

Neurodevelopment disorders can result in facial dysmorphisms. Therefore, the analysis of facial images using image processing and machine learning techniques can help construct systems for diagnosing genetic syndromes and neurodevelopmental disorders. The systems offer faster and cost-effective alternatives for genotyping tests, particularly when dealing with large-scale applications. However, there are still challenges to overcome to ensure the accuracy and reliability of computer-aided diagnosis systems. This article presents a systematic review of such initiatives, including 55 articles. The main aspects used to develop these diagnostic systems were discussed, namely datasets - availability, type of image, size, ethnicities and syndromes - types of facial features, techniques used for normalization, dimensionality reduction and classification, deep learning, as well as a discussion related to the main gaps, challenges and opportunities.

## Highlights

• Predominance of deep learning techniques since year 2000.
• Transfer learning using facial images is better.
• Average diagnosis accuracy of 90.6%.
• Influence of ethnic factors is poorly explored.
• Lack of explainability regarding facial attributes.

## 1 Introduction

Computer-aided diagnosis (CAD) systems, largely based on artificial intelligence and image processing techniques, have been used in various areas to assist physicians in the diagnostic process [1]. An area benefited from these systems is the diagnosis of genetic syndromes and neurodevelopmental disorders.

Neurodevelopmental disorders (NDDs) are a common group of conditions that first manifest as impairments early in childhood, characterized as development and function deficiency. As defined in the Diagnostic and Statistical Manual of Mental Disorders, 5th edition (DSM-5), NDDs include intellectual deficit (ID), autism spectrum disorder (ASD), attention deficit hyperactivity disorder (ADHD), communication disorders, motor disorders, and specific learning disorders [2]. In the United States, according to data published by the National Center for Health Statistics (NCHS) in 2015, an estimated 15% of children aged 3 to 17 years are affected by NDDs [3]. Diagnosis is important to delineate treatment, prognosis, and recurrence risk. NDDs often show complex patterns of impairment in the motor, cognitive, and neurobehavioral domains. There can be significant diagnostic overlap and co-occurrence of NDDs within an affected individual and/or among family members. Although the etiology of NDD is heterogeneous, genetic variation represents the largest contribution. The genetic architecture of NDDs is complex. Monogenic and polygenic cases, have been described. Although rare and common variants occur concomitantly, lower intellectual quotient (IQ), epilepsy, early motor alterations and body and facial dysmorphisms are associated with individuals with less family history, explained by the fact that very rare *denovo* variants are more frequent in those cases, but with worse prognoses [4]. Facial dysmorphisms are also present in several genetic syndromes [5] that, although rare, together affect 5% of the world population [6]. In this context, recognition of facial dysmorphisms plays a crucial role in facilitating quick and low-cost screening for a gold-standard genetic tests. However, not all physicians have been adequately trained in clinical genetics and it is not easy to scale such evaluations.

Indeed, anthropometric measurements, including craniofacial measures, have been used for years in pediatrics to detect deviations from typical development, such as general malformations or dysmorphisms typical of some genetic syndromes [7]. Measures such as landmarks distances and angles are manually obtained and compared to expected values according to genre and age [8]. With the advance of machine learning in medical informatics, the use of these techniques was a natural and fundamental step forward in order to aid diagnosis. Therefore, image processing and pattern recognition can be combined to extract features from digital facial images and thereby learn a predictive model to aid diagnosis, a CAD system.

In this area of CAD systems for NDD and genetic syndromes, the state of the art includes DeepGestalt [9], a deep learning framework trained with thousands of images that include more than 200 different genetic conditions with reported 91% top-10 accuracy. However, this framework is part of a suite of proprietary applications [10]. Facial-based CAD is a hot topic, and many works have been developed in this area. High-quality training data volume is crucial, but difficult to achieve. Therefore, many attempts to compose a successful method have emerged in order to test different techniques, features and other aspects. It is crucial to know what has been archived, as well as its corresponding results to create better strategies.

Although the literature has several primary studies on the subject, to our knowledge there is only one scoping study that compiles articles from a single search portal published until 2019 [11], with a brief survey of general aspects but not considering the influence of many factors for diagnosis performance, trends or challenges, and surveying only three works based on deep learning. The objective of this article is to present a systematic review of 55 studies published until December 2022 in five different search portals that expand the aspects analyzed in the cited work and present a quantitative and qualitative analysis of the articles included. It is also noteworthy that many of the works published from 2019 to 2022 used deep learning, which enriched the analysis on this topic in this article, considering a total of 30 articles based on deep learning. Thus, the main contributions of this work are:

• a detailed analysis of the methods used with the particularities that categorize the studies;
• a schematic diagram that categorizes/analyzes the processing phases of the approaches to promote the definition of new approaches;
• cataloging the public and private databases used, which could be reused in new studies^1^;
• discussion of gaps and challenges that configure research opportunities in the application area and in the areas developing image processing and machine learning techniques, including deep learning.

We believe that this deep analysis and discussion about the articles can help motivate and innovate the area of computer-aided diagnosis based on facial images.

## 2 Methods

The systematic review was conducted and prepared according to the PRISMA protocol (*Preferred Reporting Items for Systematic Reviews and Meta-Analyses*) [12]. The execution consisted of three steps: 1) planning the review protocol; 2) performing the searches in the chosen databases and applying the inclusion and exclusion criteria based on the titles and abstracts of the articles; 3) full reading of selected articles and extracting relevant information for summarization.

The objective of this review was to answer these main research questions:

• What methods are used to recognize neurodevelopmental syndromes or disorders from facial images?
• Are open databases available for research?
• Are there studies exploring the effects of ethnicity on diagnosis?

Firstly, an exploratory analysis was conducted using general searching engines, of which five digital libraries were the source for this review: PubMed [13], IEEE Xplore DL [14], Scopus [15], Web of Science [16] and ACM DL [17]. The search keywords were created based on the three main groups of keywords composed to identify articles that 1) are related to syndromes and disorders, 2) are based on facial images and 3) use computational techniques, as summarized in Table 1. The complete search strings for each digital library are described in Supplementary File 1. The articles from journals and conferences used in the review were those that met the inclusion criteria and did not fulfill any of the exclusion criteria, shown in Table 2. Once this work intends to review computational methods used in aiding diagnosis based on facial images, articles that just applied some software proposed elsewhere in a new image dataset were not included.

**Table 1:** Keyword groups used to create the search strings. Complete search strings are in Supplementary File 1.

Group	String
(1)	words related to phenotypes (eg. “genetic disorder”,
	“facial phenotype”, “Down”)
	**AND**
(2)	words related to images (eg.“image analysis”, “photography”,
	“facial image”)
	**AND**
(3)	words related to machine learning (eg. “machine learning”,
	“classification”, “computer aided diagnosis”)

**Table 2:** Inclusion and Exclusion Criteria.

Inclusion criterion	Description
I1	The article proposes a new framework using computational
	learning techniques to diagnose a genetic syndrome or
	neurodevelopmental disorder based on facial images.

Exclusion criteria	Description
E1	Absence of computational method/application description.
E2	Just application of a software proposed elsewhere.
E3	Absence of genetic syndrome or neurodevelopmental disorder.
E4	Not written in English.
E5	Not fully available.

**Figure 1:**
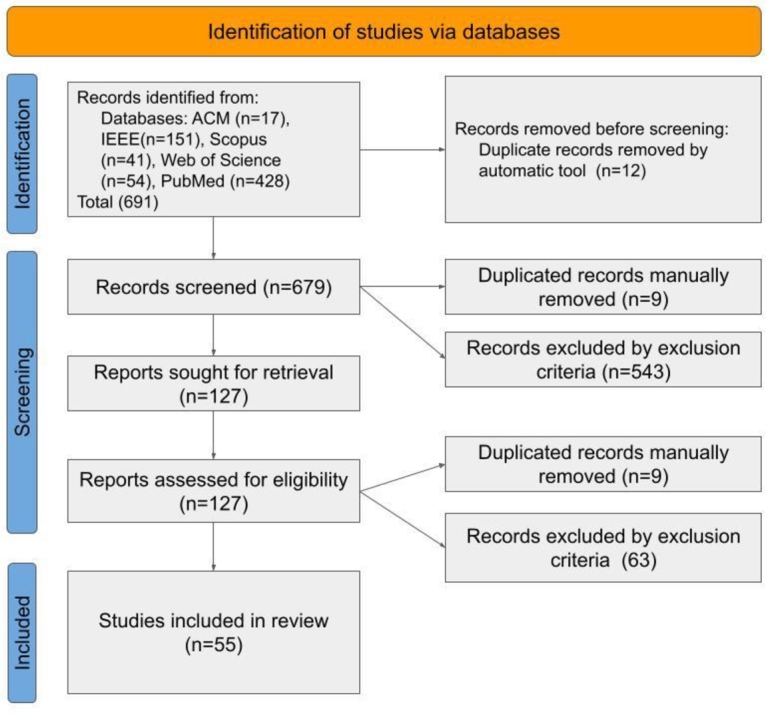
Pipeline for selecting the articles.

The systematic literature review steps are described in Fig. 1. Limiting the search for articles published until December 2022, 691 articles were returned from the five databases. After manually and automatically detected duplicates were removed and inclusion and exclusion criteria were applied, 55 studies were included in this review.

## 3 Results

The results are arranged in two sections. Section 3.1 provides a quantitative overview of the included works, while Section 3.2 presents qualitative analyses based on the works so as to present the main features and how each work is correlated within this revision. The table in Supplementary File 2 summarizes the main information of each work used in both sections.

### 3.1 Quantitative analysis

**Figure 2:**
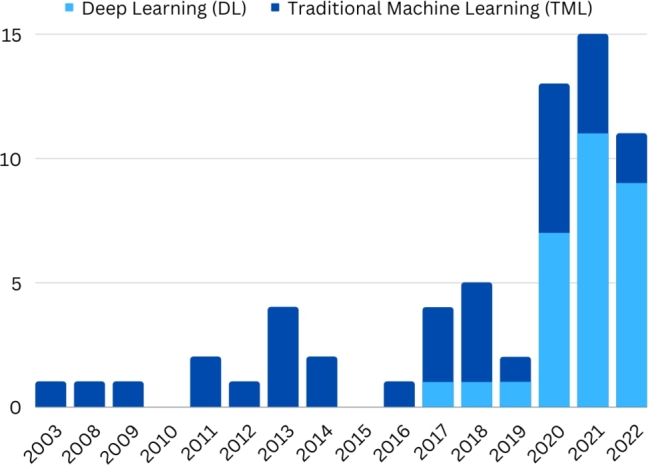
Evolution of articles published in the area.

Fig. 2 describes the selected articles distributed over the years, from 2003 to 2022. Although there are publications almost every year, it has occurred more consistently since 2016, peaking in 2021, which indicates a research increase for such systems. Deep learning has seen an increase in recent years, used initially in 2017 for extracting deep features [18] and in 2018 used initially for extraction and classification [19].

Of the selected works, 71% analyze the problem from the point of view of binary classification, considering cases that involve a disorder and a control group. However, 35% of the studies analyze more than one disorder in addition to the control group, analyzing the problem from the point of view of multiclass classification, which on average, considered 11 classes (not taking into account the study [9] that considers 216 classes). From these, three studies (6%) used both approaches. Table 3 presents the most frequent syndromes addressed by the articles. Nevertheless, the Down syndrome has an expressive volume of studies (33% of the articles). The table in Supplementary File 3 lists all syndromes and disorders studied.

**Table 3:** Syndromes and disorders studied in at least two studies.

Syndrome or disorder	No. of articles	Percentage of articles
Down	18	33%
Williams-Beuren	14	25%
Autism	11	20%
Cornelia de Lange	11	20%
Noonan	9	16%
Turner	8	15%
DiGeorge (22q11.2 deletion)	7	13%
Fragile X	7	13%
Angelman	6	11%
Fetal Alcohol	5	9%
Treacher Collins	5	9%
Acromegaly	4	7%
Progeria	4	7%
CHARGE	3	5%
Cushing's syndrome	3	5%
Kabuki	3	5%
Koolen-de Vires	3	5%
Marfan	3	5%
Prader–Willi	3	5%
Rubinstein-Taybi	3	5%
Sotos	3	5%
Wolf-Hirschhorn	3	5%
Apert	2	4%
Coffin Lowry	2	4%
Mowat Wilson	2	4%
Mucopolysaccharidosis type III	2	4%
PACS1	2	4%
Pitt-Hopkins	2	4%
Smith Magenis	2	4%
Smith-Lemli-Opitz	2	4%
Stickler	2	4%

**Figure 3:**
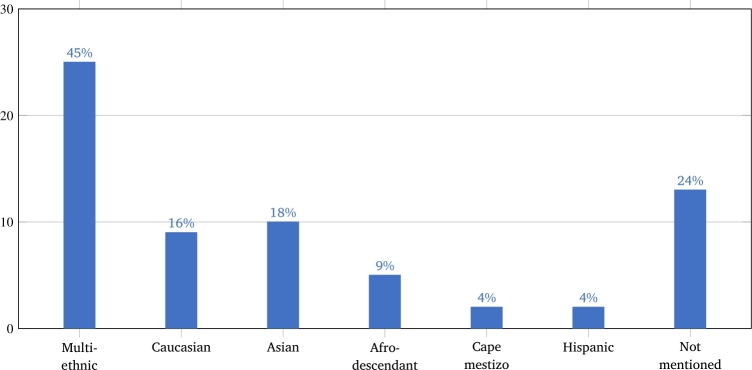
Ethnicities mentioned in the studies.

The individual's ethnicity is another concern regarding classification based on facial images. Fig. 3 shows that many studies do not even mention the ethnicity of participants (24%), and most of the studies used multi-ethnic datasets (45%). The remainder used images of individuals of a specific ethnicity, where the predominant ethnicity was Asian (18%).

**Figure 4:**
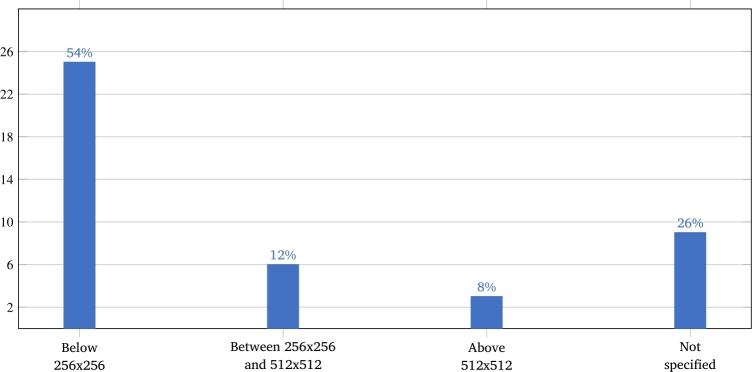
Image resolutions used in the studies.

Most of the selected works (91%) used two-dimensional (2D) images, while the rest (five works) used three-dimensional (3D) images. Fig. 4 shows how the 2D image resolutions are distributed, ranging from 80x80 to 1500x1000 pixels. Of these, resolutions below 256x256 pixels were the most used (54% of the works).

**Figure 5:**
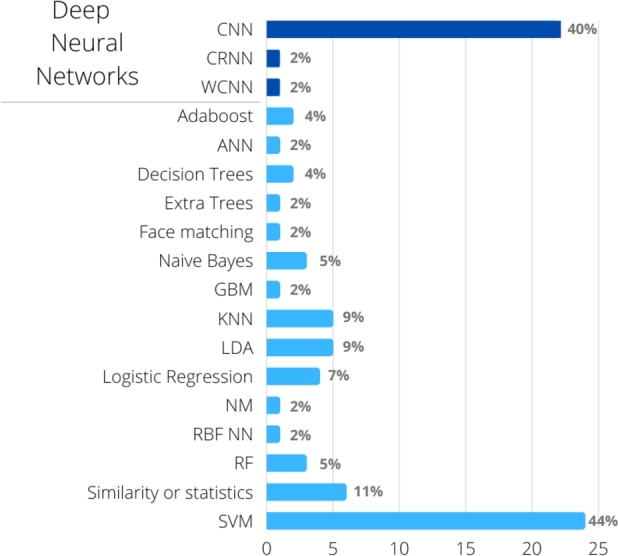
Classifier induction algorithms used in the studies. Some studies used more than one algorithm. CNN: Convolutional Neural Network, WCNN: Wide hidden layer CNN, CRNN: Convolutional-Recursive Neural Network, SVM: Support Vector Machine, KNN: k-nearest neighbors, LDA: Linear Discriminant Analysis, RF: Random Forest, ANN: (non-deep) Artificial Neural Networks, RBF NN: Radial Basis Function Neural networks, GBM: Gradient Boosting Machine.

For training the classifiers, Fig. 5 shows that the most used algorithms are those based on deep neural networks and Support Vector Machines (SVM). In addition to these, other algorithms were used, but less frequently.

**Figure 6:**
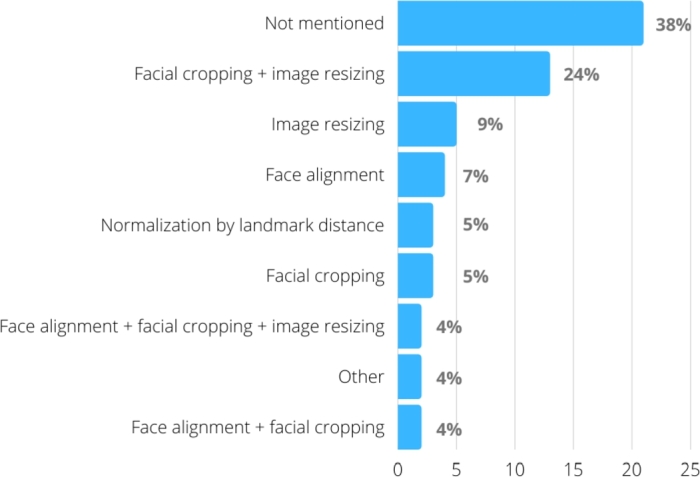
Strategies for normalizing the images used in the studies.

Another relevant issue in this type of work is to use or not image normalization strategies, especially important when photographs are captured using different distances between the camera and the face, or different zoom levels. Fig. 6 summarizes the main techniques used, which specifies their isolated or combined use, showing that the most used combination in the works was facial cropping combined with image resizing (24%).

Regarding the databases used in the studies, 58% of the works used only proprietary databases, 36% used only public databases^2^ and 6% mixed proprietary and public databases. One study [20] used images collected by indexed image search engines. However, the image set is not available.

### 3.2 Qualitative analysis

**Figure 7:**
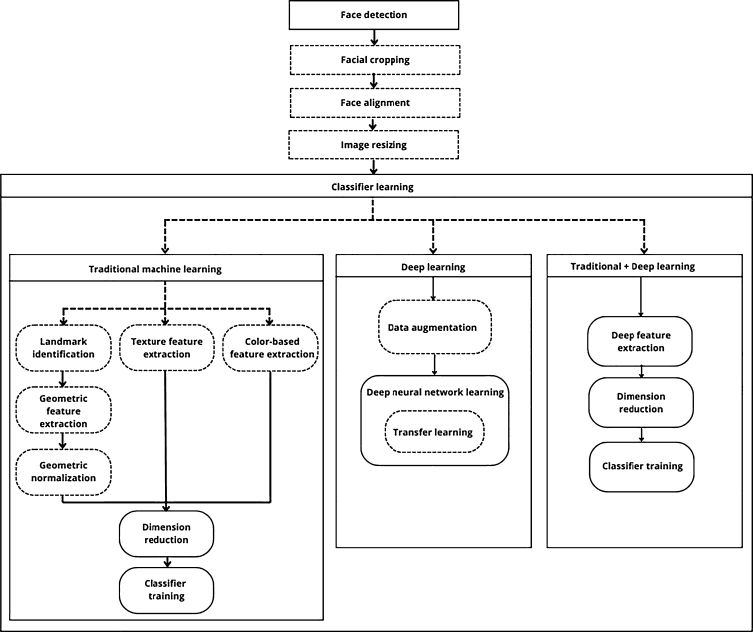
Overall classifier learning pipeline and its variations. Dashed arrows indicate alternative paths followed by articles. Processes with dashed lines are those performed by some but not all articles in that learning category.

This review includes papers that describe their methods with a sequence of processes summarized in Fig. 7. Face detection is the only and usually first preprocessing task performed in all studies. Other tasks are performed depending on the work, especially those related to some type of image normalization. Regarding the classifier learning stage, they are divided into three broad categories: 1) articles that, before the induction stage of a traditional machine learning classifier, perform a feature extraction stage that extracts geometric, texture or intensity-based features 2) articles based entirely on deep learning, with or without transfer learning and/or data augmentation, and 3) articles that use deep learning only to perform a deep feature extraction, to later learn by traditional classifier induction algorithms.

**Figure 8:**
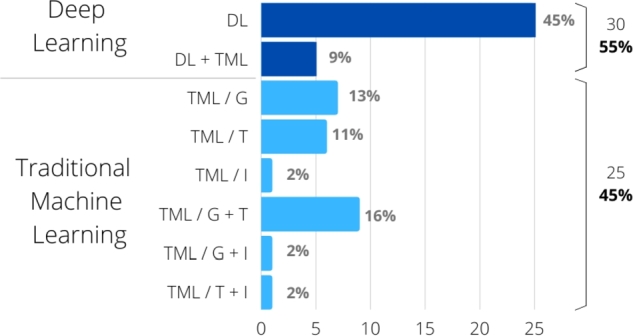
Articles distributed by category. DL: use of deep learning for feature extraction and classification; DL+TML: use of deep learning for feature extraction and traditional machine learning for classification; TML/*: use only traditional machine learning using only geometric features (TML/G), only texture (TML/T), only intensity-based (TML/I), geometric combined with texture (TML/G+T), geometric combined with intensity (TML/G+I), or texture combined with intensity (TML/T+I).

Fig. 8 depicts the articles distributed by these categories. Since articles using 2D and 3D images have substantially different methods, articles using 2D images are described in sections 3.2.1 to 3.2.3, while those using 3D images are described in section 3.2.4.

#### 3.2.1 Traditional machine learning

##### Use of geometric features only

The geometric features in this section are based on distances between landmarks detected on the face through image processing libraries.

Cornejo et al. [21] proposed a combination of pre-processing techniques, such as the use of Principal Component Analysis (PCA) and Linear Discriminant Analysis (LDA) for dimensionality reduction and differentiation between two classes (Down syndrome and control). Thus, they propose using a compact geometric descriptor based on 14 distances between facial landmarks, normalized by the face width. The algorithm of Viola and Jones was used for facial detection [22].

Kuru et al. [23] implemented a system to assist in the diagnosis of 15 syndromes, and the graphical interface is intended for the end user. PCA was used to reduce dimensionality over Euclidean distances between facial anthropometric points, and kNN as a classification algorithm, achieving an average accuracy of 60%.

In [24], a great differential is the use of frontal and lateral images. An initial step regards the application of the Gabor wavelet transform, which results in the construction of 96 anthropometric points, and applies the PCA to select the first 70 components. Multinomial logistic regression was used for classifier induction. Of the eight experiments performed, the best results were those that combined frontal and side images.

The mix of features from the use of different tools is evaluated by van der Donk et al. [25]. The authors combined the feature vectors extracted by two different tools, CFPS (*Clinical Face Phenotype Space*) and OpenFace, into a hybrid feature vector, which were then analyzed with statistical and clustering techniques to identify rare genomic neurodevelopmental disorders.

Distance computation is a key factor included in the approaches. Practically every study presents pre-processing techniques to reduce the dimensionality. Furthermore, all studies cite the libraries and tools that extract features and locate landmarks.

##### Use of texture features only

In this category the studies extract from the two-dimensional input images only features based on the detected face texture. The texture features can represent the relationships of a pixel with its neighbors in a two-dimensional image, offering a way to identify patterns. So, when a two-dimensional image represents a three-dimensional object, identifying different levels of luminosity, it contributes to recognizing light and dark areas related to the object's different levels of depth. This process highlights relevant differences related to the face components that can be carried out among individuals, but not detected if only geometric features extracted from landmarks are considered – as shown in the previous section. Shape of nose and mouth, for example, is captured in 3D images, but texture features are necessary to capture them when input images are obtained and processed considering the two-dimensional domain.

Loos et al. [26], the oldest article in this review, used only 55 images from patients with five different syndromes: Mucopolysaccharidosis type III, Cornelia de Lange, Fragile X, Prader–Willi, Williams–Beuren. Fourty texture features were calculated from each image, with a set of Gabor wavelets of different spatial sizes and orientations, which are represented by a bunch graph. Using a leave-one-out strategy, the test image was automatically labeled using elastic graph matching and similarity comparison with the other 54 bunch graphs. This method showed a 76% accuracy, whereas clinical geneticists who were shown the same photographs achieved 62% accuracy.

The use of LBP and DLBP (*Derivative Local Binary Pattern*) was also analyzed by Tabatabaei and Chalechale [27] for comparing techniques, and the use of DLBP showed a small improvement in the results.

Schneider et al. [28] proposed an automatic classifier for acromegaly recognition that displayed greater performance to that obtained by acromegaly experts and general internists using only visual impression. The experiments were carried out using the FIDA^3^ software, combining Gabor jets to extract texture features and construct a graph, based on the control points. The classification is based on the *Elastic Bunch Graph Matching* application, which consists of calculating the similarity between the graphs.

Applying a very similar approach, Kosilek et al. [30] presented a classifier aimed at Cushing's syndrome in women. Despite the small sample size (20 cases and 40 age-matched controls), they achieved 91.7% accuracy. The same group expanded the sample (82 cases and 98 controls) and included both genders in [31], this time using controls corresponding to cases that not only used age and gender, but also body mass index. In the second study, these controls were patients with suspected syndrome but who had negative diagnoses. This choice of sample composition was probably what caused the classification accuracy to decrease to the 60% range.

The work by Dudding-Byth et al. [32] uses texture-based facial recognition software to identify *matches* of the input face with facial images from ten databases of different syndromes. The classification is then performed for the syndrome that has the image with the highest *match*, or that takes into account the five or ten images with the highest *match*.

##### Combination of geometric and texture features

The aforementioned articles extract the two types of features from the faces detected in the two-dimensional images and use them as input for training the classifier.

The work proposed by Zhao et al. [33] introduces techniques not previously applied in this area of study, such as *Constrained Local Model* (CLM) combined with PCA during the process of automatic detection of 81 facial anthropometric points. The points were used by the authors to extract 24 geometric features including angles and vertical and horizontal distances calculated from landmarks, all chosen from clinical knowledge of Down syndrome. The horizontal distances were normalized by the distance between the external points of the eyes, and the vertical distances by the vertical distance between the eye line and the base of the lower lip. In addition to geometric features extracted from these points, the authors also extracted texture features using LBP in the pre-processing of the model input set, which had good results with a reduced volume of attributes. It is also noteworthy that, initially, each face had been aligned with a reference face, using Procrustes analysis, to eliminate translation, rotation and scale change. In this work the authors also compare the use of SVM, kNN and *Random Forest* as classifiers.

These same approaches were improved twice by these authors [34] and [35], who proposed using a tangled ensemble of pairs of classifiers based on SVM, *Random Forest* and linear discriminant analysis (LDA), which increased the performance measures. A classifier ensemble is also used in [36], but executed a face alignment and gloss normalization in the pre-processing stage.

The work presented by Cerrolaza et al. [37] addresses a possible approach to apply a technique very similar to LBP for facial identification. Although there is no in-depth detailing of the *pipeline* development and execution stages, the classification inducer obtained accuracy results above 90%, which may present the combination of geometric and texture features as a very promising technique to solve this problem.

Chen et al. [38] showed that using facial local features can be more effective than global ones. They used 212 female participants (54 patients with Turner Syndrome and 158 controls) to compare SVM classifiers, trained with only global geometric features (GGF) or with only global texture features (GTF), with an AdaBoost classifier trained with only local geometric and texture features (LF): epicanthus, melanocytic nevus, ocular distance, forehand, and nasal bridge. Results using local features and AdaBoost were better: sensibility of 67%, 44% and 23% for LF, GTF and GGF, respectively, and all with at least 80% specificity.

For aiding Cornelia de Lange syndrome diagnosis, Dowsett et al. [39] used geometric features based on distances and angles calculated from facial landmarks, and texture features based on local binary patterns. Support Vector Machine classifiers were trained using a multi-ethnic set of images from individuals from 15 countries. The global accuracy, estimated by leave-one-out cross-validation, was of 94%, whereas for Latin American, African descent, Asian, and Caucasian groups the accuracies were of 98%, 96%, 95%, and 95%, respectively.

The same machine learning strategy was applied in [40] and [41], that also investigated the ethnic influence. In [40], an important result was the accuracy differences which were obtained while training classifiers with more or less specific ethnic groups: 91.67% using Democratic Republic of the Congo, 77.27% using African descent and 76.76% using global population for Down syndrome diagnosis. In [41], the aim was the discriminating between Noonan and Williams–Beuren syndromes, which share similar facial phenotypes, using images from patients from 14 countries, including African descent, Asian, Caucasian and Latin American population. Using a global model trained with all individuals, the global accuracy was of 85.68%, varying from 83.23% (for Caucasians) to 87.30% (for African descents). However, using ethnicity-specific models, the global accuracy improved to 90.38%, varying from 87.88% (for Asians) to 93.65% (for African descents).

##### Use of intensity-based features

In this work, we state that a feature is based on intensity when it is based on the pixel value variation. Zhao et al. [42] used only this type of feature to assist in the diagnosis of Turner syndrome. PCA and kernel-based PCA were applied based on the pixel vector of the gray level images with subsequent selection of features using the Minimum Redundancy - Maximum Relevance (mRMR) algorithm. Unlike the other works, they used ensemble learning to create the classifier, testing majority voting and stacking strategies to combine several SVM classifiers.

Dima et al. [43] explored the use of Eigenfaces, which can be defined in general terms as a combination of PCA and LBP (*Local Binary Pattern*), to attenuate the most expressive signs and subsequently reduce noise from the application of the Wavelet transform. This strategy resulted in good performance results, although it is a more extensive and complex processing flow than most of the analyzed works.

#### 3.2.2 Articles that largely use deep learning

This section describes the articles that do not perform a separate step of extracting previously established features because they use deep learning.

Since deep neural networks involve learning many parameters, the end result will be compromised if the training sample is small. Transfer learning is a technique often used to circumvent this problem. It consists of using a deep neural network trained in a similar domain with a large training sample, and additional layers are then added, whose parameters will be learned from samples of the specific problem, a step known as fine-tuning. For syndrome diagnosis, the last layers are trained with labeled samples of the syndromes in question. Regardless of whether transfer learning is used, data augmentation can also be executed. Which is when training images are used to create new variant images, for example, changing lighting or applying rotations. Of the 12 works described in this section, nine applied data augmentation and nine used transfer learning, eight of which combined both techniques. Of those that use transfer learning, six use previously established models and three train their own models.

##### Transfer learning with pre-established models

The articles here described used pre-established deep neural network architectures – pre-trained or training them in a general dataset – and then fine-tuned them with images of case and control subjects for classification. Table 4 summarizes the accuracies achieved. Articles using the Kaggle-ASD dataset for fine-tuning were grouped together to allow comparing the results using the same or different datasets.

**Table 4:** Accuracy achieved using the several pre-established models and training datasets. The models are presented in ascending order of number of parameters. IN: ImageNet, VF: VGGFace, YF: YoutubeFace, CWF: CASIA-WebFaces.

Articles		[44]	[45]	[46]	[47]	[48]	[49]	[50]	[51]	[52]	Kaggle dataset avg. acc.	[19]	[53]	[54]	[55]	[56]	[57]	[58]	Global avg. acc.
Sample size for fine-tuning		2654	2654	2540	1269	2536	2556	2538	2936	2283		1240	12986	262	365	1911	1549	238	
No of classes		2	2	2	2	2	2	2	2	2		12	3	5	35	2	2	2	
Kaggle-ASD Dataset?		yes	yes	yes	yes	yes	yes	yes	yes	yes		no	no	no	no	no	no	no	
Pre-established models (No. of parameters in millions)	Training dataset																		
MobileNet-V2 (3.5M)	IN	94.6%	95%		88.6%	84.7%		87.5%			90.1%							85.6%	89.3%

InceptionResNetV1 (5M)	CWF										-					90.7%			90.7%

EfficientNetB0 (5.3M)	IN					86.3%					86.3%								86.3%

NASNETMobile (5.3M)	IN			78%							78%								78%

EfficientNetB1 (7.8M)	IN					89.7%					89.7%								89.7%

EfficientNetB2 (9.2M)	IN					88.7%					88.7%								88.7%

ResNet18 (11.7M)	IN						76%				76%							87.9%	82%

ResNet34 (22M)	IN										-							89.1%	89.1%

Xception (22.8M)	IN		94%	91%		90%	74%				87.2%								87.2%

ResNet50 (23M)	IN							66%	86%		76%	97.5%		77.3%			-		81.7%
	VF										-			82%			-		82%
	YF										-						-		-

Inception v3 (23.9M)	IN		89%		85.2%						87.1%		95%						89.7%

DenseNet161 (28.6M)	IN						87%				87%								87%

ResNet101 (44.6M)	IN						87%		85.3%		86.1%								86.1%

InceptionResNetV2 (56M)	IN				98.2%						98.2%								98.2%

ResNet152 (60M)	IN								87%		87%								87%

AlexNet (61M)	IN										-			66%					66%
	VF										-			73.3%					73.3%

VGG-16 (138M)	IN						83%	82%	89%		84.7%			72%				90.9%	83.4%
	VF									95%	95%			86.7%	88%				89.9%

VGG-19 (144M)	IN			80%					88%		84%							92.7%	86.9%

Wei et al. [53] were the first to apply deep learning to classify Acromegaly, Cushing's syndrome and control group. For transfer learning, they used the Inception v3 model pre-trained from ImageNet dataset. In fact, ImageNet is a set of general images and not just faces, that is, from a very generic domain. Even so, they obtained 95% of accuracy, probably due to the large size of the training sample: more than 14 thousand images after having applied data augmentation.

Singh and Kisku [19] use the weights from the pre-trained VGGFace model and apply them to the ResNet50 architecture. To perform the fine-tuning, they tested two approaches: four and three fully connected final layers, and for the configuration with three layers an SVM classifier was also added on top. After data augmentation, both configurations were trained with 1772 facial images from 12 classes of genetic syndromes, achieving similar accuracies and close to 97%.

Jin et al. [54] compared the pre-trained AlexNet, VGG16 and ResNet networks on the ImageNet and VGGFace datasets using two approaches: (i) fine-tuning the networks and (ii) using them as a feature extractor for an SVM classifier. The best result was obtained with the VGG16 architecture, pre-trained with the images from the VGGFace dataset, used as a feature extractor for a linear SVM classifier, reaching an accuracy of 93% despite the small sample size (70 images of each syndrome without mentioning data augmentation).

Hong et al. [55] also used the pre-trained VGG-16 architecture in the VGGFace dataset so as to classify 35 different genetic syndromes. Fine-tuning was performed using augmented data from 456 original images. The accuracy of the model was 88%, higher than the accuracies achieved by five pediatricians.

Kong et al. [56] not only proposed diagnosing acromegaly but also classifying the severity of the disorder. The authors used the Inception ResNet v1 network and pre-trained with the Casia-WebFace dataset images and used it as a feature extractor. On top of it they added two layers of branches, one based on softmax loss and the other on center loss, ultimately combined for total loss optimization. Fine-tuning was performed from 2148 images, after data augmentation, achieving 90.7% accuracy.

Fu et al. [57] tested the training of ResNet50 architecture with VGGFace2, ImageNet and a Youtube Face subdataset. In addition, they focused on developing four regularization strategies during fine-tuning, which include restricting resource outputs from the middle and classification layers, which were applied to classify children with fetal alcohol syndrome. The best result was achieved by the training performed with the VGGFace2, with fine-tuning performed from augmented data from 1549 original images.

Liu et al. [58] tested five different deep CNN architectures (VGG-16, VGG-19, ResNet-18, ResNet-34, and MobileNet-V2) using transfer learning from ImageNet and fine-tuning the neural networks with 340 images from Williams-Beuren Syndrome and control individuals (healthy and with other syndromes). The accuracy obtained by these architectures increased in the same direction as the increase in their number of parameters. The highest accuracy (92.7%) was obtained by VGG-19 whereas the lowest accuracy (85.6%) was obtained by MobileNet-V2. To compare these results to human performance, the authors invited four human experts to classify the images. And the best human accuracy was 82.1%.

The next nine articles [44], [45], [46], [47], [48], [49], [50], [51], [52], published from 2021 to 2022, used a public dataset of 3,014 internet-collected images of potentially healthy children or potentially with Autism Spectrum Disorder, added in the Kaggle platform on 2020 [59] (currently no longer available - see discussion in section 4.3).

Hosseini et al. [44] used a pre-trained model, MobileNet, but added three extra network layers for fine-tuning: one global average-pooling and two dense layers with 124 and two neurons, obtaining accuracy of 94.6%.

The remaining eight works compared different pre-trained deep network architectures, fine-tuning and testing them with images from this ASD dataset. MobileNet-V2 was the most used (five articles using this dataset), presenting a good average accuracy (90.1%), whereas InceptionResNetV2 presented the highest accuracy (98.2%). Both averages were calculated based only on Kaggle ASD dataset results (Table 4).

Two of these studies deserve additional comments: [51] that used automatic machine learning, and [52] that discussed ethnic factors.

In [51], five pretrained models, a CNN without transfer learning, ten traditional machine learning methods and Automatic Machine Learning (AutoML) are compared. AutoML, which automatically trains, optimizes hyperparameters and evaluates different models, achieved the highest accuracy (96%) with Keras slim residual neural network classifier. For non automatically trained deep learning classifiers, accuracies varied from 84% (CNN without transfer learning) to 89% (VGG16). For non automatically trained traditional ML classifiers, accuracies varied from 55.6% (Logistic Regression) to 72.6% (Extra trees). These results show the importance of exploring the AutoML strategies that are able to evaluate several different ML algorithms, saving human effort and processing time, while achieving better results due to its optimization methods.

Lu and Perkowski [52] used the same Kaggle ASD dataset only to illustrate the influence of ethnic factors in ASD classification based on facial images. First, The VGG-16 pre-trained model was fine-tuned with 2283 reliable images derived from several sources from China and East Asia, all from Asian children, which achieved 95% accuracy. Next, the Kaggle dataset, composed of approximately 11% African descent and 89% Caucasian images, together with the Asian dataset, was used to investigate the influence of ethnic factors on the classification performance. In fact, training and testing with the same dataset (Kaggle), shows that the false positive rates differ in ethnic groups: 15% for Caucasian and 75% for African descent children.

Comparing the performance of all pre-trained models, using or not the Kaggle-ASD dataset, Table 4 shows that many factors may influence the results: the model used, the dataset for fine-tuning, and mainly the dataset used to train these models (transfer learning). It is perceived that models pre-trained with ImageNet are correlated to accuracies lower than those achieved by models pre-trained with VGGFace. This can be explained by the fact that ImageNet is a database of general images whereas VGGFace is completely composed of facial images, a domain that is much closer to the target domain of facial-based diagnosis. This factor was more decisive than, for instance, the number of parameters of the models.

Most of the articles developed binary classifiers, but those using higher number of classes also obtained good results (Table 4). Indeed, the second highest accuracy (97.5%) was reported for the classifier with 12 conditions, using a moderate size fine-tuning dataset [19]. Also impressive is the 88% accuracy obtained in [55] using a moderate size fine-tuning dataset (365 images) for 35 classes.

##### Proposal of new network architectures and use of transfer learning

Instead of using pre-established models for facial recognition, four articles [9], [60], [61], [62] created their own network architectures, trained them with images from CASIA-WebFaces [63] - a dataset containing almost 500 thousand facial images, and finally, performed fine-tuning with specific images for the problem domain.

Gurovich et al. [9] proposed the DeepGestalt *framework*, which is the basis of the Face2Gene software. A DCNN is created, first for facial recognition. Fine-tuning was performed with a usually large volume of training images: more than 17 thousand images that represent more than 200 syndromes, reaching an accuracy of 90%.

After training with the Casia-WebFaces dataset, the DCNN proposed by Qin et al. [60] was fine-tuned using 405 images so as to differentiate between Down cases and controls, it reported an accuracy of almost 96%. The authors highlight the approach for overfitting correction and for the optimization of parameters of the deep convolutional neural network (DCNN), based on the increase in the number of layers in the convolutional networks.

Yang et al. [61] proposed a CNN that has a differential known as loss function, called additive angular margin loss (ArcFace). The aim is to identify Noonan syndrome in children. After training with the Casia-WebFaces dataset, the network was fine-tuned using Noonan images (127x4), other dysmorphic syndromes (130x4) and controls (163x4), where x4 means that for each original image, another three were obtained by data augmentation. The reported accuracy was 92%.

Wang et al. [62] developed a residual neural network adding Squeeze-and-Excitation (SE) blocks to enhance the weights of valid regions and weaken the weights of invalid regions. The classifier achieved 93.5% accuracy.

##### Articles that did not use transfer learning

Although some works did not apply transfer learning, they used relatively small training samples.

Hababeh et al. [20] used a convolutional neural networks of wide hidden layers (WCNN) to identify individuals with Down syndrome. This wide architecture is characterized for having more neurons in the hidden layers than a high number of layers with fewer neurons. The training was performed using 5128 images, after data augmentation, achieving 88.97% precision.

Porras et al. [64] developed a deep learning technique structured in three neural networks, one for image standardization, another for detecting of facial morphology, and the last for estimating the risk of genetic syndrome, taking into account phenotypic variations due to age, sex and ethnicity. The networks were trained with approximately 22 thousand images obtained from data augmentation of 2800 original images. The result for the total population was an average 88% accuracy, with similar results across ethnicities, age groups and genders.

Niu et al. [65] proposed a neural network composed of convolutional and residual layers based on ResNet34, fine-tuned with 1832 images that contained patients with Turner syndrome and controls. They achieved 97% accuracy, a value higher than those obtained with other techniques also tested in this article: MLP trained with geometric, texture and color features, and two other deep network models: VGG16 and ResNet18.

Arumugam et al. [66] trained a new CNN architecture with the Kaggle dataset of potentially healthy children or with autism, achieving 91% accuracy (estimated using one holdout).

Pan et al. [67] trained a ResNet-based CNN for Turner Syndrome (TS) diagnosis using 170 photographs of TS patients and 1053 photographs of controls, obtaining an average 96% accuracy. The authors also tested the impact on the classification performance while training models using age matched or not age matched samples, with no significant difference found. The same happened for height matched or not height matched samples.

#### 3.2.3 Articles that combine traditional and deep learning

Although deep neural networks perform both feature extraction and classification tasks, one possible approach is to use these networks only for feature extraction (deep feature extraction) and then use them as input for a traditional induction algorithm of classifiers. This was the approach used by the works described in this section. All of them also used transfer learning, and none of them mention the use of data augmentation.

Shukla et al. [18] proposed using five pre-trained CNNs (Alex-Net model) for feature extraction, each one responsible for analyzing the total image of the face or just one of four parts (top right, top left, bottom right or bottom left), to explore global and local face features. The features they extract are unified into a single feature vector used to train a linear SVM classifier. The authors analyze six syndromes in four experimental scenarios: (1) all syndromes together vs. control (binary classifier); (2) each of the six syndrome classes vs. control (six binary classifiers); (3) a multiclass classifier to differentiate syndromes and (4) all syndromes together vs. control, but all from the same age group (three binary classifiers, one for each age group). In scenario 1, they reached 98.8% performing training with a sample of about 1800 images.

Liu at al. [68] proposed an unsupervised feature extraction technique in which CRNNs (Convolutional-recursive neural networks), a combination of CNN with RNN, are used for pre-training filters and to create high-level features. Therefore, after clustering image patches using the Kmeans algorithm, they were used to train convolution filters that generate feature maps, which are then processed by multiple recursive neural networks to generate the image features. Afterwards, SVM is used to classify the images. The authors achieved 84.95% accuracy in the classification between Turner syndrome cases and controls, with a total training sample of 628 images.

The next two articles [69], [70] used, in addition to the conventional 2D deep features, 3D geometric features in different ways.

Although Geremek et al. [69] used the 3D Analyses Face Alignment Network to extract 16 3D landmark points and 11 distances from the original 2D images, the best results were obtained using directly the 2D images directly. Seven pre-trained CNN models were used to extract face embeddings: VGG-Face, Facenet, Facenet512, OpenFace, DeepFace, DeepID, and ArcFace. In both cases (using 2D or 3D features) SVM classifiers were trained in the context of multiclass classification (15 syndromes and controls) and binary classification (syndromes and controls). For the multiclass problem, the accuracy achieved using 3D features was only 56%, whereas the highest accuracy using 2D deep features was 84% using ArcFace. For the binary problem, the 3D and the best 2D (DeepFace) accuracies were 67% and 96%, respectively.

Kumov and Samorodov [70] also used 3D geometric features and 2D deep features, but they also tested a combination of both for the classification of eight syndromes. *Deep 3D Face Reconstruction* library was used and geometric features were extracted from the 3D reconstruction points, and the deep features were also extracted using VGG16 from the 2D images. Only geometric, only deep and combined features were applied to a sequential combination of techniques using PCA and/or LDA for dimension reduction, and classifier induction algorithms kNN, SVM, *Random Forest*, LDA, Logistic Regression and Naive Bayes. The best results (92.5% accuracy) were achieved using a combination of all deep and geometric features, PCA+LDA and Logistic Regression, working with a dataset of 1462 images.

Altogether, these last two works indicate that only 3D geometric features did not outperform the use of 2D deep features, at least when these features are extracted from 3D reconstructions from originally 2D images. Better results were obtained using 3D images, as described in the next section.

#### 3.2.4 Articles that use three-dimensional images

Five of the articles included in this review use three-dimensional images captured by a specific system for this purpose.

Fang et al. [71] used 3D laser scanning to obtain 3D images, from which they extracted features such as area, mesh aspect ratio, degree of plainness and curvature from the three-dimensional meshes. Using the *Correlation-based Feature* (CBF) and *Radial basis function networks* (RBFN) algorithms, they created a classifier for diagnosing fetal alcohol syndrome. They also compared the performance of the classifier when it was trained and tested on two separate and combined ethnic populations, which resulted in accuracies of 90% and 80%, respectively.

Wilamowska et al. [72] used 3D surface head images for diagnosing DiGeorge syndrome. Different sets of control images were evaluated, the one composed of images where each control matched each of the affected individuals of same ethnicity, sex and closest age was associated with the highest F-measure of the classifier. Geometric and intensity features were explored in the evaluation of three 3D data representations: 3D snapshots, 2.5D depth images, and curved lines, and the last one showed the best results (82% accuracy). In all representations, PCA was applied to select the top 10 components that were input to various classifier induction algorithms.

Suttie et al. [73] used 3D images captured by a commercial photogrammetric camera to classify the severities of fetal alcohol spectrum disorders. With more than 25 thousand facial landmarks from these images, they performed PCA and cluster analysis, and analyzed the differences, proposing a technique based on face signature graphs and heat map. The results obtained with LDA and SVM were also compared. The study presents a detailed analysis of facial components and anthropometric regions that have significant differences between syndrome and control, and their degrees of severity.

Meng et al. [74] captured facial images from three different angles and used them to build a single three-dimensional image for each individual of the 62 acromegalic patients and 62 controls. Then, 35 landmarks were extracted from each 3D image and used to perform a two-way analysis of variance (ANOVA) with gender (male/female) and disease condition (patient/control) as factors, a t-test between the patient and the control groups, and to train Linear Discriminant Analysis classifiers on each gender separately. The results showed that acromegalic patients are significantly different from normal subjects in many variables, and that facial changes of male patients are more significant than female ones. The classification accuracy, estimated by leave-one-out cross-validation, was 92.86% for females and 75% for males.

Whereas the works based on 3D images, mentioned so far, used only traditional machine learning techniques, Mahdi et al. [75] used geometric deep learning (GDL) to extract features directly from the non-Euclidean facial surfaces for classification of 13 different syndromes. In addition, part-based and full-face approaches were tested. Both approaches compared the performance of LDA classifiers trained with GDL and PCA features. The best results achieved used the part-based approach. In both approaches, GDL features improved the classification performance when compared to PCA features.

## 4 Discussion

The next sections in this systematic review highlight our main considerations on many of the aspects evaluated in the articles. We also consider probable research opportunities and challenges.

### 4.1 3D and 2D images

Only 10% (5 papers) of the reviewed articles used 3D images captured by a special equipment, published in 2008, 2009, 2013, 2020 and 2022. Due to the continuous improvement in the quality of 2D images captured by common digital cameras, all other articles were based on this type of image. The advantages of 2D images regard the democratization of data collection due to the need for more affordable equipment, and a greater availability of images, since many of the datasets used were created from photographs captured by the family. This high quantity of images was key for using classification techniques based on deep learning, as discussed in the next section.

It is noteworthy that only one work used deep learning on 3D images, given that this field is still under development. Traditional CNN architectures are designed for 2D input images. Instead, [75] used spiral convolutional operators on a non-Euclidean domain. Although [70] also used deep learning and 3D reconstruction, the deep features were extracted from the 2D original images.

### 4.2 Extracted features used in traditional machine learning methods

Geometric features were extracted in 33% of all articles. These features were calculated based on facial landmarks detected by image processing libraries such as Dlib [76], OpenCV [77] and OpenFace [78]. A critical point that should be highlighted is that most of these libraries use well-known models trained to detect landmarks, and several of them are repeatedly cited as a proven good solution to identify these points. Nevertheless, no study reported evaluating these tools in a pre-processing step. Considering that these models usually present limited generalization capacity, it is a risk to assume that their performance is high for all datasets the results. Validate the performance of these models could be a good practice, as reported in [79] and [80] and, if necessary, retrain them with the dataset in use.

Texture features were extracted in 31% of all articles. The are two important points in this group of articles: (1) there is no trend in the features extracted, and (2) there is considerable variation in the size of datasets and results obtained. Regarding the former, the existing libraries that provide reusable implementations have again favored the use of different features. Obviously, this could be encouraging because it can increase the productivity in developing the CAD; however, implementing different features can hinder comparing the approaches. Usually researchers do not justify their choice of a group of texture features nor compare different features to solve the same problem. This can therefore lead to the latter point: variation of the dataset size and characteristics of the datasets (resulting in various results), reinforces the impossibility of comparing them. These points could be overcome if researchers from similar areas concentrated their effort to work together to build public datasets that can adequately validate different approaches and, hence, contribute towards meaningful progress in the state-of-the-art. This is particularly important for approaches based on texture features, since the projection of three-dimensional object (the human face) in two-dimensional images can generate an enormous variety of patterns that only very big datasets could include sufficient samples to represent different ethnicities, gender and ages. This is essential to build efficient approaches in different scenarios.

Only two articles (4%) specifically extracted intensity-based features, and all were based on gray-level of the pixels. The use of pixel color values was not observed with traditional machine learning classifiers. In fact, the use of colors can introduce a new difficulty when processing facial images. First, there are different color models to represent images. Although conversion between different models is possible, there may be some data loss. Second, this can add a processing step that requires more computational resources. Third, ethnic characteristics and other aspects such as ambient lighting can generate different color tonalities that the algorithms interpret as different information. Lastly, gray level images are usually enough to extract geometric and texture features, which include a large amount of information about the facial components. Maybe these topics justify limiting the explicit use of color-based features. It is important to mention that, in the context of deep learning, CNNs attempts to introduce in their models the use of convolutional filters that can implicitly consider the color. These approaches have favored the use of color images when using deep learning.

Two types of features, mostly geometric and texture features, were extracted in 20% of the articles. Again, this category of studies shows variation in the features, datasets and results. Considering the advance of the hardware and the availability of libraries, we observe a trend for extracting as many features as possible from different categories and, afterwards, use strategies to reduce the dimensionality. The positive aspect of hybrid approaches is the complementary of information, since geometric descriptors provide data not covered by texture descriptors and vice versa. However, this trend can generate noise in the process, decreasing the models' performance. When AI conventional techniques are used, we think the most efficient approach is to expand the problem knowledge and define features that represent the experts' knowledge whenever possible, avoiding unnecessary computational processing, while also minimizing the probability of including useless data that could confuse the models.

### 4.3 Classification techniques and dataset sizes

As highlighted in section 3.1, Fig. 2 shows the prevalence of traditional machine learning techniques in articles published until 2018, when techniques based on deep learning became predominant. Deep learning needs massive amounts of data to train the large number of parameters that comprise the models. In fact, the articles published until 2018 worked with datasets of 558 images on average. In this review, the oldest article [26] used a total of 55 images for five syndromes, while the first article using deep learning [18] used 2252 images (six syndromes and controls). Considering the whole period (from 2003 to the end of 2022), articles using traditional machine learning were able to classify syndromes with datasets of 421 images on average, with results often comparable to those based on deep learning that had 3,088 images on average, often applying data augmentation, and not counting the images used for transfer learning when applied. The most extreme example was when 17,106 syndrome and control images were used, in addition to the nearly 500,000 facial images used for transfer learning [9]. At the other extreme, the smallest dataset used with deep learning techniques was composed of 350 images, however using a pre-trained network for transfer learning [54]. Therefore, research carried out with traditional machine learning methods should not be neglected in problems that have a small available dataset. From these traditional methods, SVM was by far the most widely used (Fig. 5), which raises the question whether other methods ought to be more explored, such as boosting.

Transfer learning and data augmentation were frequently used by deep learning algorithms: 73% and 37% of the articles in this category, respectively, and 30% used both strategies. For transfer learning, most of the articles used pre-trained models (Table 4). The most used was the MobileNet-V2 (89.3% average accuracy), and the best result was achieved using InceptionResNetV2 (98.2% accuracy). Some articles trained their own architectures, and few of them did not use transfer learning. In this case, the smallest training set used for classifier training had 1223 images [67] and 628 images for feature extractor training [68], both for binary problems (case and control).

It is well known that dataset size is an important issue to better train the models. However, dataset labeling quality is equally important. So building big high-quality dataset is a challenging issue, particularly as it involves a careful diagnosis of individuals by specialists. Therefore, care should be taken with less reliable datasets. For instance, in this review seven articles used the Kaggle Autistic Children Facial Image Data Set (Kaggle-ASD) [59]. However, this dataset is composed of internet-searched, low-quality, low-fidelity images. Among the problems, such as the presence of varied facial expressions, pose variation and dirty labeling (presence of other syndromes) [81], there is no guarantee of the class labels since the images were collected from the internet instead of being captured from individuals evaluated by health professionals in a controlled context.^4^ In fact, Kaggle removed this dataset. Although results using this dataset seem satisfactory, the results obtained in [52] raise some questions. The testing done on a high-quality dataset of Asian children shows the false positive rate increasing from 6.67% when the model was trained in this Asian dataset to 86.73% when the model was trained in the Kaggle-ASD dataset. Since Kaggle-ASD is primarily composed of Caucasian children, is the ethnic influence the only explanation for this difference? Or does the quality difference provide part of the answer? No article compared Kaggle-ASD with a high-quality ethnic-matched ASD database to help us answer these questions.

### 4.4 Pre-processing, normalization and resolution of images

Regardless of the classification technique used, face detection is a pre-processing task, usually the first one, performed in all works. The other works change the application and combination of techniques, mainly those related to some type of normalization so that the images are comparable (Fig. 6), despite the fact that 38% of the works do not explicitly mention this concern.

Since works based only on traditional machine learning perform a previous feature extraction step, they often use normalizations related to the nature of these features. For example, geometric features can be normalized by certain landmark distances [21]. On the other hand, works based on deep learning normally need same-size images to be used as input for the networks. Thus, facial cropping followed by image resizing are often used. Furthermore, to process the high data volume necessary for this type of learning, face-cropped images are resized to reasonably modest sizes, which explains the predominance of images with resolutions below 256x256 pixels (Fig. 4).

Other techniques are also applied, such as Procrustes analysis [33], [34], [35]. Such analysis allows aligning and removing the scale component of the distances, since the mean squared distance of the points is normalized. In other words, it becomes a statistical measure of the scale or distance size.

In fact, works entirely based on traditional deep learning, after extracting and potentially normalizing the features, often apply dimensionality reduction steps using techniques such as principal component analysis (PCA) [23], linear discriminant analysis (LDA) [21] or other feature selection algorithms [42].

### 4.5 Lack of explainability or level of importance regarding facial attributes

Few works used interpretable methods or presented which features had the most impact to result in a given classification.

Boehringer et al. [24] showed how specific elements of the syndromes under analysis can be visualized in relation to themselves and the control group, and evaluated the areas that may contain zones of uncertainty in the classifier.

Suttie et al. [73] presented the construction of a graph based on similar features, which offered a different perspective of analysis. A correlated view was presented to the patients, in addition to the insertion of a heat map on each middle face to help visualize the regions of interest of these components.

Another highlight is the work by Zhao et al. [35], which presented a classification of features by relevance based on clinical aspects for Down Syndrome.

The authors of [40] tried to explain the impact of each selected feature estimating its individual discriminative power using the non-parametric Mann-Whitney U test.

With the increased use of deep learning from 2020, explainability was even less explored. This is an issue that should be addressed, since the “trusty artificial intelligence” theme is a hot topic and a currently pursued goal [82]. This issue is particularly important in the context of aiding diagnosis to avoid misclassifications or biases of any kind (ethnic, age, etc.). Therefore, interpretable machine learning methods should be more explored or, when using non-interpretable methods such as deep learning, agnostic interpretability techniques should be added in the frameworks [83].

### 4.6 Gaps, challenges and potential opportunities

One of the research challenges in developing classifiers for the diagnosis of syndromes and disorders is the poor comparability of studies, making it difficult to identify what is really more effective in the area. Although many of the studies reported their results in terms of accuracy (82% - see Supplementary File 2), the results were achieved with different datasets, and in general with different syndromes. Most datasets are proprietary (58% of the works), particularly those that are well-characterized, in which participating volunteers have undergone professional hospital screening [55]. There are also datasets created in a less systematic way, for example collected by image search engines on the internet [20]. There are few initiatives to make available curated data, such as the Collaborative Initiative on Fetal Alcohol Spectrum Disorders (CIFASD) [84], whose data were used by two studies included in the present work [57], [71]. Therefore, the proposal and availability of public benchmarks could improve comparing the studies, which would allow an actual analysis of the most effective techniques for each problem studied. Considering the legal and ethical issues involved in the creation of public datasets, an alternative approach to allow a fair comparison between different methods would be to make available the codes that composed the approach, which would also favor verifying the generalization capacity of the approaches based on the execution with new datasets. However, software availability was very rare in the reviewed articles.

Generally, there was no concern with class imbalance, that is, the difference between the proportions of images of each syndrome and controls within the training datasets. A few studies collected a number of control images equal to the number of case images they had [33], [27], [21], and the rest did not mention any posterior balancing strategy such as those based on oversampling or undersampling, widely used in traditional machine learning [85]. However, it is expected that such a balance will require more attention when using deep learning techniques, in which the images themselves are used as input to the networks. One possibility would be to apply data augmentation to balance the proportions. Data augmentation techniques have been applied with some deep learning constancy, performing operations such as rotation, translation, change in brightness and contrast, among other operations. Image augmentation techniques per se is a research theme in deep learning [86], even using other deep neural networks dedicated to these activities, such as generative adversarial networks (GANs) [87]. Within the context studied here, a research opportunity could be investigating the best techniques [88], [89].

Another little explored issue in the studies was the analysis of the effect of training segmentation and classification on ethnic, age, gender and severity groups. Few studies have addressed these issues, and have not evaluated all of them [40], [41], [56], [64]. Regarding ethnicity (Fig. 3), the lack of attention to this issue is evidenced by the fact that 24% of the articles did not even mention ethnicity in the dataset, and that multiethnic datasets were the most frequent (45%). It would be of interest to investigate whether ethnic-specific classifiers could be more accurate than the general ones, as well as to carefully assess the results obtained with general classifiers when applied to an ethnic group that is very different from that used mostly in training. To what degree ethnicity-specific classifiers are better than multi-ethnic ones may depend on the specific ethnic group. For instance, while the authors [41] improved the accuracy by 9.7% at most, when creating ethnicity-specific models for African descent, Asian, Caucasian and Latin American populations (in the context of Noonan and Williams–Beuren syndromes discrimination), the authors of [40] improved the Down syndrome classification accuracy by 15% (from 76.76% to 91.67%) using only Democratic Republic of the Congo individuals instead of global population. Ethnicity has been shown to highly impact the performance of Face2Gene in the classification of Down syndrome patients of Caucasian and African origin [90]. Regarding gender, fewer studies explored this issue. The authors of [74] found an accuracy difference of almost 18% between male and female acromegalic patients. Considering that some conditions have a gender-specific prevalence [91], gender-specific models could be explored.

Most of the studies based on deep learning used CNN architectures. However, other types of deep networks and additional advances in artificial intelligence can be explored, both for feature extraction and classification. Some examples are deep belief networks [92], Multi-level Graph Learning Network (MGLN) [93], MLP_Mixer [94] as well as hybrid architectures [95].

Finally, although recent years have seen substantial growth in the area of deep learning, a somewhat larger data volume for training is needed, which is highlighted in this review. However, there are rare syndromes and disorders whose training sample size (on the order of 4 to 16 images [55], [25]) usually leads to not-so-good results even when using transfer learning. Traditional machine learning techniques could be explored in such cases. Most of the studies using these techniques reported accuracies above 90%, even with small samples. However, SVMs were used in 50% of these works, while other classifier induction algorithms were little explored (Fig. 5). Some little explored strategies were ensemble learning [35] – which could provide more robust classifiers, and automatic machine learning tools – used on only one article [51].

## 5 Conclusions

This article presented a systematic review of studies that used computational methods to construct classifiers of genetic syndromes or neurodevelopmental disorders from facial images. The evolving degree of sophistication embedded in solutions that increasingly seek a high degree of effectiveness has been observed in recent years. Interestingly, the most used approaches are hybrid, the datasets are predominantly proprietary and, with the expanding data availability, deep learning data is more widely used. These features of the analyzed set of articles create challenges and, consequently, research opportunities, such as the elaboration of techniques and datasets to compare the different approaches, techniques to produce balanced datasets and to deal with datasets that are still very small. Hopefully, the outcomes in this work regarding the methods used and related aspects can help advance this area so that these systems can be developed quicker and can diagnose those syndromes and disorders so as to apply more large-scale reliable screening tools.

## CRediT authorship contribution statement

Fábio Rosindo Daher de Barros, Caio Novais F. da Silva, Gabriel de Castro Michelassi, Helena Brentani, Fátima L. S. Nunes, Ariane Machado-Lima: Analyzed and interpreted the data; Wrote the paper.

Fábio Rosindo Daher de Barros, Helena Brentani, Fátima L. S. Nunes, Ariane Machado-Lima: Conceived and designed the analysis.

Fábio Rosindo Daher de Barros, Caio Novais F. da Silva, Gabriel de Castro Michelassi, Ariane Machado-Lima: Contributed analysis tools or data.

## Declaration of Competing Interest

The authors declare that they have no known competing financial interests or personal relationships that could have appeared to influence the work reported in this paper.

## Data Availability

Data included in supplementary material.

## Appendix A Supplementary material

MMC 1 Search strings used in the review process to search for articles. MMC 1

MMC 2 Information extracted from each included article. MMC 2

MMC 3 A list of all syndromes addressed in the articles and the total number of times each one was discussed. MMC 3

## Footnotes

1 This and additional information is in Supplementary File 2.

## Footnotes

2 Public databases were those with public access or those that need asking the authors through e-mail or by filling out a form.

3 FIDA or (*Facial Image Diagnostic Aid*) is a software that applies a set of techniques to classify disorders and syndromes through similarities [29].

4 According to the author of the dataset, Kaggle removed this dataset claiming violation of their terms of service [59].
